# Poly(carboxylate ether)-based superplasticizer achieves workability retention in calcium aluminate cement

**DOI:** 10.1038/srep41743

**Published:** 2017-01-30

**Authors:** Omid Akhlaghi, Yusuf Ziya Menceloglu, Ozge Akbulut

**Affiliations:** 1 Faculty of Engineering and Natural Sciences, Sabanci University, Istanbul, 34956, Turkey

## Abstract

Calcium aluminate cement (CAC) suffers from loss of workability in less than an hour (~15 minutes) after first touch of water. Current superplasticizers that are utilized to modify the viscosity of cement admixtures are designed to target ordinary Portland cement (OPC). The high affinity between these superplasticizers and cement particles were found to be detrimental in CAC systems. Utilization of a monomer that, instead, facilitates gradual adsorption of a superplasticizer provides workability retention. For the first time in literature, we report a superplasticizer that caters to the properties of CAC such as high rate of surface development and surface charge. While neat CAC was almost unworkable after 1 hour, with the addition of only 0.4% of the optimized superplasticizer, 90% fluidity retention was achieved.

CAC is the cement of choice for high performance applications such as those requiring resistance to abrasion, corrosion, and temperature^1^,^2^,^3^. Due to its high early heat and strength gain, CAC has also become an attractive binder for cold environments and situations that necessitate rapid repairs (e.g., highways, bridge decks, and airport runways). In general, long term durability of cementitious mixtures requires low water/cement ratio (w/c). At low water contents, superplasticizers, which are polymers that can facilitate the dispersion of cement particles, are utilized to provide necessary workability. Superplasticizers adsorb onto the surface of the cement particles through their charged backbone and provide electrostatic repulsion^4^,^5^. They release the entrapped water from flocculated structures and modify the viscosity of cement mixtures^6^,^7^. Latest generations, poly(carboxylate ether)-based superplasticizers, (PCEs), have acrylate groups in the backbone and also contain side chains (i.e., poly(ethylene oxide)) that protrude from the cement surface into the pore solution to produce steric hindrance effect^8^,^9^. These grafted polymers exhibit superior dispersing ability compared to other types of superplasticizers (e.g., melamine and naphthalene based polycondensates) and are efficient in the preparation of high performance concrete^5^,^10^,^11^.

The formulations of PCEs have always targeted OPC since it is the most frequently used binder in cement industry. However, these superplasticizers are incompatible with CAC due to i) the rapid hydration reaction of monocalcium aluminate as the principal active phase in this type of cement and ii) layered structure of hydration phases in CAC compared to amorphous calcium-silicate-hydrate (C-S-H) in OPC^6^,^12^,^13^. The use of PCEs in CAC systems resulted in poor fluidity retention (~15 min) and intercalation/sequestration of PCEs into lamellar calcium aluminate hydrates^13^,^14^. Due to the absence of CAC-optimized superplasticizer, CAC systems are utilized in their neat form and consequently lose their workability in a relatively short amount of time^15^. Therefore, for wider and more efficient use of CAC, there is a need for the design of superplasticizers that can accommodate the characteristic properties of CAC.

Here, we report a modified PCE-based superplasticizer that can address the underlying mechanisms of hydration kinetics, development of surface area, and surface charges in CAC systems. To incorporate into the acrylic acid (AA) backbone of PCEs, we have chosen two co-monomers i) 2-acrylamido-2-methylpropane sulfonic acid (AMPS) and ii) vinylphosphonic acid (VPA), with different ionic character and ability of association with multivalent cations. AMPS exhibits strong ionic character^16^ whereas VPA demonstrates strong complexation ability with divalent cations^17^,^18^. To comprehend the criteria of adsorption and workability retention, we cross-compared the performance of superplasticizers that contain varying amounts of AMPS and VPA in both OPC and CAC systems. The effect of both co-monomers on electrokinetic behavior of cement suspensions and rheological response of cement pastes were explored to design a dedicated superplasticizer for CAC. The fast reaction kinetics of hydrating CAC can be accommodated by controlled adsorption of VPA-modified PCEs (VPA-PCEs) that leads to the workability retention of the cement paste for longer periods of time (>1 hour). This type of PCEs is the first set of superplasticizers to enable fluidity retention of CAC systems.

## Result and Discussion

Eight PCEs with increasing amounts of modifying co-monomer in the backbone, referred to as X5, X10, X20, and X30 (X: VPA, AMPS) were prepared^19^. All of the copolymers contain PEO1000 side chains with a density of less than 1.1%. The general chemical structure of polymers and molar ratios of building blocks, determined by H^1^-NMR (Supplementary Figures S1A and S1B), were shown in Supplementary Figure S2 and Supplementary Table S1, respectively.

### Adsorption behavior and electro-kinetic study

**Figure 1:**
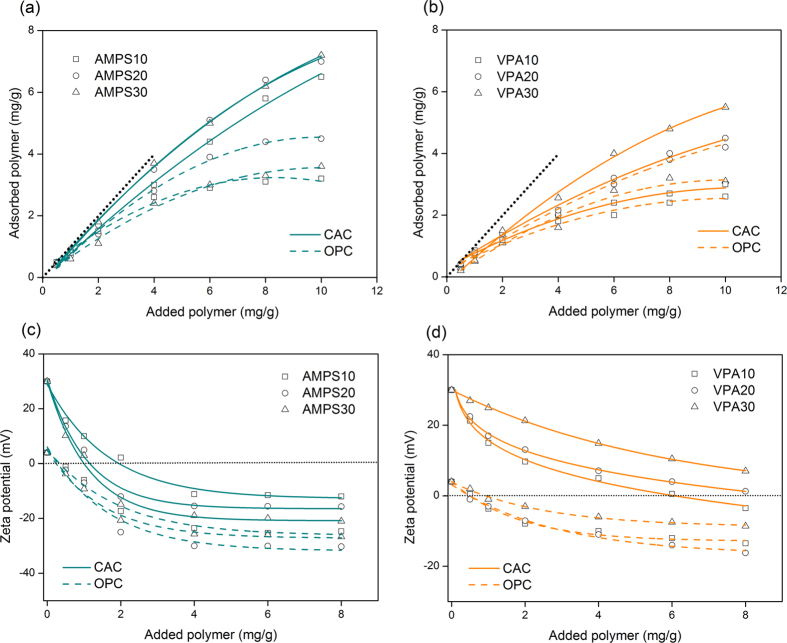
Adsorption behavior of (**a**) AMPS-PCEs and (**b**) VPA-PCEs on the surface of cement particles as a function of polymer dosage (dotted lines show 100% adsorption). Zeta potential of cement suspensions in the presence of (**c**) AMPS-PCEs and (**d**) VPA-PCEs.

The dispersing ability of PCEs depends on the adsorption of the copolymer onto the cement particles. Adsorption of PCEs typically follows the Langmuir monolayer model while multi-layer adsorption is plausible at high dosages of PCEs^20^,^21^,^22^. We first tracked the adsorption of modified-PCEs through measuring the amount of unadsorbed copolymer that remains in the solution (depletion method)^10^. Once adsorption starts, the amount of adsorbed PCEs in AMPS-PCEs/OPC, VPA-PCEs/OPC, and VPA-PCEs/CAC systems increases linearly with the amount of added polymer at low dosages (Fig. 1). The adsorption, then, stabilizes to a plateau value (i.e., adsorption saturation) confirming the Langmuir monolayer adsorption behavior of PCEs in these systems. This plateau indicates complete coverage of cement particles by PCEs while the slope of the linear range is related to the affinity of PCEs to the cement particles^14^. VPA-PCEs exhibited lower adsorption tendency compared to AMPS-PCEs in both OPC and CAC systems; whereas utilization of anionic co-monomer (AMPS-PCEs) caused depletion of the PCEs from CAC suspensions. To elucidate the effect of modifying block on induced charge of the cement particles upon adsorption of PCEs, zeta potentials were evaluated at differing amounts of polymer in the cement suspension. In general, interactions of PCEs and cement particles comprise i) the electrostatic interactions and ii) formation of complexes between the Ca^2+^ and the ionic backbone of PCEs^21^,^23^. Unlike direct electrostatic adsorption of PCEs, adsorption through Ca^2+^ bridging has little influence on the zeta potential of cement particles^22^. AMPS co-monomer adsorbs onto the surface of the cement particles through strong conjugation in its sulfonate group (SO_3_^−^)^24^. Compared to carboxylic group of AA, which is a weak acid with strong complexation ability, sulfonic group is a stronger acid and mainly interacts with the surface of the cement particles via electrostatic interactions^25^. Lower basicity of the oxyanion in SO_3_^−^ compared to that of acrylate (COO^−^) reduces the charge transfer to counterions and results in ionic character of its bonding with counterions^24^. On the other hand, phosphonate groups (PO_3_^2−^) present more basic oxyanions than COO^−^ and its bi-functionality, compared to mono-negative charge of COO^−^, gives rise to strong complexation with multivalent cations^17^,^18^. Therefore, substitution of AA with AMPS co-monomer encourages the shift of surface potentials to higher negative values whereas VPA substitution is expected to impart a slight change on the surface charge of the particles.

**Figure 2:**
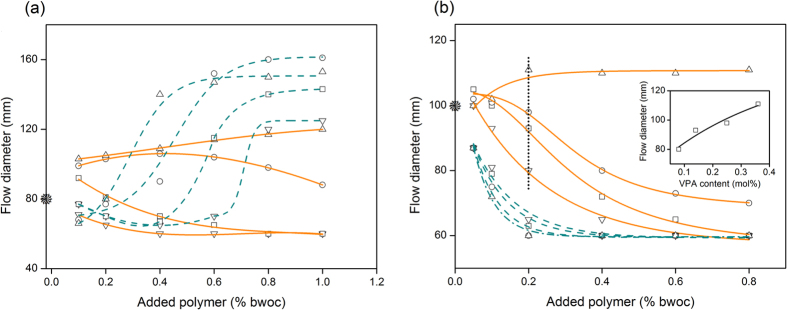
Fluidity behavior of (**a**) OPC and (**b**) CAC pastes in the presence of AMPS-PCEs (dash-line) and VPA-PCEs (solid line), (∇): X5, (□): X10, (○): X20, and (Δ): X30. Solid star shows flow diameter of neat cement pastes. Inset of Fig. 2b shows flow diameter of CAC pastes in the presence of 0.2% VPA-PCEs as a function of VPA content in the backbone of copolymer.

In hydrating OPC, the presence of negatively charged silicate and positively charged aluminate phases leads to the formation of heterogeneous charge distribution on the surface of particles^26^. Compared to OPC, CAC, whose main component is monocalcium aluminate, offers higher positive zeta potential and thus, more anchorage points for direct adsorption due to the fast reaction of the aluminate phase at early stages of hydration^3^. Upon adsorption, zeta potential of suspensions decreased from +4 mV in neat OPC and from +30 mV in neat CAC (Fig. 1c and d) confirming that negatively charged PCEs progressively consume positive charges on the surface of the particles. PCEs, once adsorbed, bring more negative charges to the surface than needed to compensate all of the positive ones. Therefore, increasing amount of adsorbed PCEs eventually leads to an inversion of the zeta potential (overcharging effect)^27^. In OPC systems, the decrease in zeta potential ceases at 6‒8 mg/g of PCEs that is consistent with the dosages where the particles are fully covered (Fig. 2a and b). After full coverage of OPC particles with AMPS-PCEs, overcharging effect is clearly observed where zeta potential lowers below −20 mV. However, in CAC, surface of particles offers more positively charged areas; thus, it is less susceptible to overcharging effect. As a result, inversion of zeta potential appears at higher dosages and zeta potential cannot go below −15 ± 4 mV in CAC systems.

To further understand the role of surface charge density on instability of CAC suspensions, surface area of the neat cement in the first 5 minutes of hydration (during mixing process) was determined (Supplementary Figure S3). In early stages of CAC hydration, unequal solubility of Ca^2+^ and aluminate ions leads to roughening of the surface and enrichment of Al_2_O_3_. This incongruent solubility of ions increases the surface area and charge density of the hydrating particles, whereby double hydroxides of cationic [Ca_2_Al(OH)_6_]^+^ are formed as the hydrating product^28^,^29^. In agreement with the results of Mangabhai^29^, we have also observed high rate of surface development in CAC suspensions (Supplementary Table S2) that is accompanied by the introduction of high positive potentials to the surface of particles (+30 mV). Therefore, high rate of surface development, surface charge density of hydrating particles, and affinity of adsorption result in depletion of anionic PCEs (AMPS-PCEs) from CAC suspensions. In VPA-PCEs/CAC systems, the highest amount of phosphonate substitution (VPA30) resulted in i) the least overcharging effect (i.e., no inversion in the sign of zeta potential) and ii) the highest Ca^2+^ complexation as demonstrated by Ca^2+^ titration and conductivity measurements (Supplementary Figure S4). In agreement with the less ionic character of phosphonate group compared to that of carboxylate group, these observations illustrate that Ca^2+^ mediated adsorption is the dominant process for VPA-PCEs/CAC systems. Therefore, through the utilization of phosphonic groups, adsorption affinity of PCEs to the surface of CAC particles is lowered and the change of surface potential is restrained upon adsorption.

### Flow behavior and workability retention of cement pastes

Flow behavior of cement pastes depends on the physical and chemical interactions among its components: cement particles, admixtures (e.g., superplasticizers and stabilizing agents), and water^12^. Generally, the adsorption of superplasticizers on cement particles deflocculates the aggregate structure of the fresh paste and releases the restrained water and therefore, gives rise to an improved fluidity of cement mixtures^30^. To assess the compatibility of superplasticizers and cement, we carried out a mini slump test as a function of dosage of PCEs^31^. In this test, a cone of a height of 60 mm, and bottom and top diameter of 40 mm and 20 mm, respectively, is filled with cement paste and spread diameter is recorded after pulling out the cone. Typically, the flow diameter of cement mixtures increases with low concentration of the superplasticizers, and then, reaches a plateau at a certain dosage (i.e., critical dosage). Beyond this critical dosage, fluidity of the mixture does not depend on the amount of superplasticizer since the dispersion state of particles does not change with further addition of the superplasticizer^21^. On the other hand, incomplete surface coverage of particles below a “minimum dosage” might decrease fluidity of the mixtures due to inhomogeneity in charge distribution and broad range of surface potentials^31^. Critical and minimum dosages were tracked to understand the effect of ionicity of the polymers on the fluidity of cement pastes. In AMPS-PCEs/OPC systems, as the content of AMPS is increased in the backbone, the minimum dosage shifted from 0.6% by weight of cement (hereafter %) in AMPS5 to 0.1% in AMPS30, and the critical dosage changed from 0.8% in AMPS5 to 0.4% in AMPS30 (Fig. 2). Moreover, minimum dosages overlap with the dosages that surface potentials of particles enter into the electrostatically stable region (<−20 mV, Fig. 1c) and particles experience homogeneous charge distribution (Supplementary Figure S5). Concurrently, critical dosages coincide with the beginning of the plateau region where surface of particles are fully covered by PCEs (Fig. 1a and c). With rise of AMPS content of PCEs, the flow diameter of mixtures at critical dosages has increased to 120‒150 mm compared to flow diameter of 80 ± 3 mm in neat OPC paste. Observed correlation between fluidity of OPC pastes and magnitude of zeta potentials in AMPS-PCEs/OPC systems indicates that electrostatic repulsion capacity of the PCEs plays a key role in dispersability of these anionic superplasticizers^32^,^33^. In VPA-PCEs/OPC systems, due to reduced affinity of adsorption and lower ionicity of VPA compared to AMPS, adsorption of VPA-PCEs cannot impart enough charge onto OPC particles (Fig. 1d). Lower fluidity of VPA-PCEs/OPC systems compared to that of AMPS-PCEs/OPC confirms that PCEs with higher anionic character are more favored for OPC paste for enhanced fluidity and compatibility.

**Figure 3:**
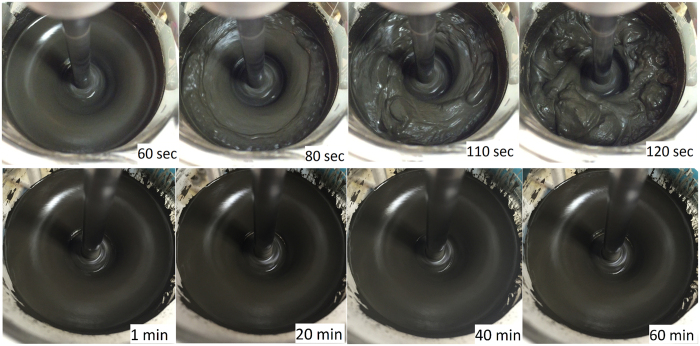
Top: quick setting of CAC paste in the presence of 0.5% AMPS30 and bottom: fluidity of CAC paste in the presence of 0.5% VPA30.

In AMPS-PCEs/CAC systems, the flow diameter of cement paste is decreased at all dosages (Fig. 2b) such that increasing ionicity of the AMPS-PCEs progressively lowers the fluidity of the pastes. As shown in Fig. 3, destabilization and quick setting of CAC paste underscores the incompatibility of CAC and a PCE-based copolymer with high anionic character. This incompatibility originates from high rate of surface development (Supplementary Table S2) and high surface charge density of CAC particles (Fig. 1c) that rapidly deplete AMPS-PCEs from the pore solution (Fig. 1a). It is important to note that zeta potentials of larger than 20 mV (or smaller than −20 mV) are typically preferred for stable suspensions^34^,^35^. Hence, AMPS-PCEs whose dispersing ability mostly relies on electrostatic interactions cannot form stable CAC suspensions even after full coverage of the particles. On the other hand, reduction of ionicity of PCEs by incorporation of VPA into the backbone progressively improves the fluidity of CAC pastes (dotted line in Fig. 2b) and led to observation of a critical dosage at 0.2% of superplasticizer in VPA30. Enhanced compatibility of CAC with VPA30 (Fig. 3) underlines that adsorption of PCEs through electrostatic interactions are detrimental for fluidity of CAC pastes. On the other hand, utilization of co-monomers with strong complexation ability facilitates the dispersion of CAC particles. In these systems, the fluidity behavior of the paste does not follow the zeta potential but rather directly correlates with the amount of adsorbed superplasticizer. Therefore, steric repulsion dominates the deflocculation of the dispersion^36^,^37^,^38^.

The dispersion state of CAC particles in the presence of VPA30 and AMPS30 is also tracked by measuring the average particle size (d_ave_) and particle size distribution (PDI) of CAC suspensions (Supplementary Figure S6A–F). In the presence of VPA30, d_avg_ and PDI decreased slightly illustrating colloidal stability of the system whereas the size of flocs increased from 3.9 ± 0.5 μm in neat CAC suspension to 6.3 ± 1.3 μm in the presence of AMPS30 (Supplementary Figure S7, Table S3). This increase in floc size demonstrates the formation of assembled structures (Fig. 3) and hence, higher amount of entrapped water in flocculated particles^21^.

To quantitatively evaluate the effect of modifying co-monomers and thus, dispersion state of particles on the fluidity of mixtures, rheological measurements were carried out on cement pastes. By fitting the experimental points of descending part of shear rate-shear stress curve (Supplementary Figure S8) with Bingham equation (τ = τ0 + η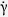), two parameters are tracked; i) yield stress (τ_0_, Pa) as a measure of the shear stress required to initiate flow and ii) plastic viscosity (η, Pa.s) as a measure of material resistance to flow after the initiation of the flow^39^. While yield stress is proportional to the particle‒particle interactions in cement mixtures; plastic viscosity relates to the size of the flocs and varies with particle size distribution in cement particles^40^,^41^. Compared to neat CAC paste, VPA30 reduced the yield stress more than 35% (Supplementary Tables S4 and S5). Introduction of AMPS to PCEs progressively reduces the yield stress and plastic viscosity of OPC pastes (Supplementary Table S4) such that more than 80% reduction of yield stress was measured in the saturation dosage of AMPS30. This pronounced effect of anionic PCEs is fully linked with higher dispersing ability of these PCEs and narrow size distribution of flocs in OPC paste. However, this increasing ionicity has an adverse effect on CAC suspensions such that rheological parameters of CAC pastes could not be assessed as all of the AMPS-PCEs/CAC pastes showed severe coagulation upon addition of these anionic superplasticizers.

**Figure 4:**
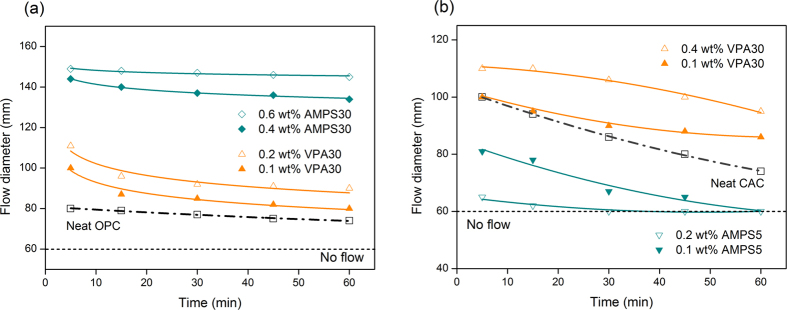
Time dependent fluidity of (**a**) OPC (**b**) CAC pastes in the presence of PCEs with highest dispersing ability.

Increase of anionicity of PCEs has been shown to enhance the adsorption rate of superplasticizers onto OPC particles and hence, initial workability of the system^42^. However, retaining this induced fluidity depends on the gradual adsorption of PCEs from the pore solution^43^,^44^. OPC, which has lower surface charge compared to CAC, can sustain gradual adsorption of the copolymer. On the other hand, anionic copolymers (AMPS-PCEs) got immediately adsorbed onto the CAC particles due to high surface development and charge in CAC systems. This depletion of AMPS-PCEs from the pore solution reduces the dispersing ability of this set of superplasticizers^13^. Therefore, achieving high initial fluidity and workability retention in CAC necessitates a controlled adsorption onto the particles. To evaluate the time dependent workability (fluidity retention behavior) of cement pastes, superplasticizers with the highest dispersing ability in each system were chosen and fluidity of the system was measured with varying dosages of the PCEs over a period of 60 min (Fig. 4). After addition of VPA30 to CAC and OPC pastes, fluidity retention in both systems was clearly improved. In VPA30-CAC system that contains 0.4% superplasticizer, only a 9% decrease in flow diameter was observed after 60 min. In the absence of PCEs, both CAC and OPC showed ~50% reduction in flowability after 60 min while approaching to the line of “no flow” at a diameter of 60 mm.

In summary, in widely used OPC systems, high affinity between the superplasticizer and the cement particles provides fluidity and stable dispersions. However, in CAC systems, gradual adsorption is necessary in order to avoid depletion of the superplasticizer from the suspension and accommodate the high surface charge and increasing surface area of the hydrating CAC particles. We utilized a co-monomer with less ionic character but with strong complexation ability, VPA, to offer controlled adsorption of PCEs and for the first time in literature, demonstrated workability retention of CAC pastes compared to the neat CAC systems. We believe this result will potentially open up venues for wider and more efficient use of calcium aluminate cement.

## Method

### Materials

Ordinary Portland cement (OPC) CEM 1 42.5 R and CAC ISIDAC 40 were provided by, AKCANSA and CIMSA, Turkey, respectively. Physical and chemical properties of cements were listed in Supplementary Table S6. Acrylic acid (AA 99%), 2-acrylamido-2 methylpropanesulfonic acid (AMPS, 99%), and potassium persulfate (KPS, ≥99.0%) were obtained from Sigma-Aldrich. Poly(ethylene oxide) (PEO1000) and vinylphosphonic acid (VPA, 97%) were purchased from Merck and Euticals, Germany, respectively. All of the reagents were used as received without further purification.

### Characterization methods

Mini slump test was carried out at 22 ± 2 °C according to ASTM C143. Average value of two crossing spread diameters after 2 times repetition of each test was reported as the test result. Adsorption behavior of PCEs to cement grains was evaluated via UV-vis spectroscopy (UV-3150 Shimadzu spectrometer). Suspensions with w/c of 2:1 and different amounts of PCEs were prepared. After 15 min of stirring, suspensions were centrifuged at 10000 rpm for 10 min and amount of adsorbed admixture was quantified by measuring the difference between UV adsorption of supernatant and bulk concentration of polymer before addition of particles. A zeta potential analyzer (Zetasizer ZS, Malvern Instruments), which has a 633 nm red laser, was used to monitor the electrokinetic behavior of particles in the presence of different amounts of PCEs. The analyzer provides zeta potentials that were calculated by determining the electrophoretic mobility of particles after applying Smoluchowski approximation. The suspensions with w/c of 40 and PCEs/cement of 0‒8 mg/g were mixed by a magnetic stirrer for 10 min, afterwards dispersed by a bath sonicator for 3 min, and finally stirred magnetically for a further 2 min. This high w/c (compared to actual w/c ratio) was selected to eliminate the background noise in zeta potential measurements^26^,^37^,^45^. According to Plank and Gretz^26^, the results of zeta potential analyses are comparable in both high and low w/c ratios. It is worth mentioning that upon addition of CAC and OPC particles to water, pH of the medium increased to ~11 and 12, respectively in less than 10 sec. The average of 6 measurements with at least 12 runs each was reported as the test result.

## Additional Information

**How to cite this article**: Akhlaghi, O. *et al*. Poly(carboxylate ether)-based superplasticizer achieves workability retention in calcium aluminate cement. *Sci. Rep.* **7**, 41743; doi: 10.1038/srep41743 (2017).

**Publisher's note:** Springer Nature remains neutral with regard to jurisdictional claims in published maps and institutional affiliations.

## Supplementary Material

Supplementary Information
