# Supplementary material for: Poly(carboxylate ether)-based superplasticizer achieves workability retention in calcium aluminate cement
Source: Sci Rep. 2017 Jan 30;7:41743. doi: 10.1038/srep41743 (PMC5278384; doi:10.1038/srep41743)
Supplement: Supplementary Information [file srep41743-s1.pdf]

Supporting Information

Poly(carboxylate ether)-based superplasticizer achieves workability retention in calcium aluminate cement

Omid Akhlaghi, Yusuf Ziya Menceloglu, and Ozge Akbulut*

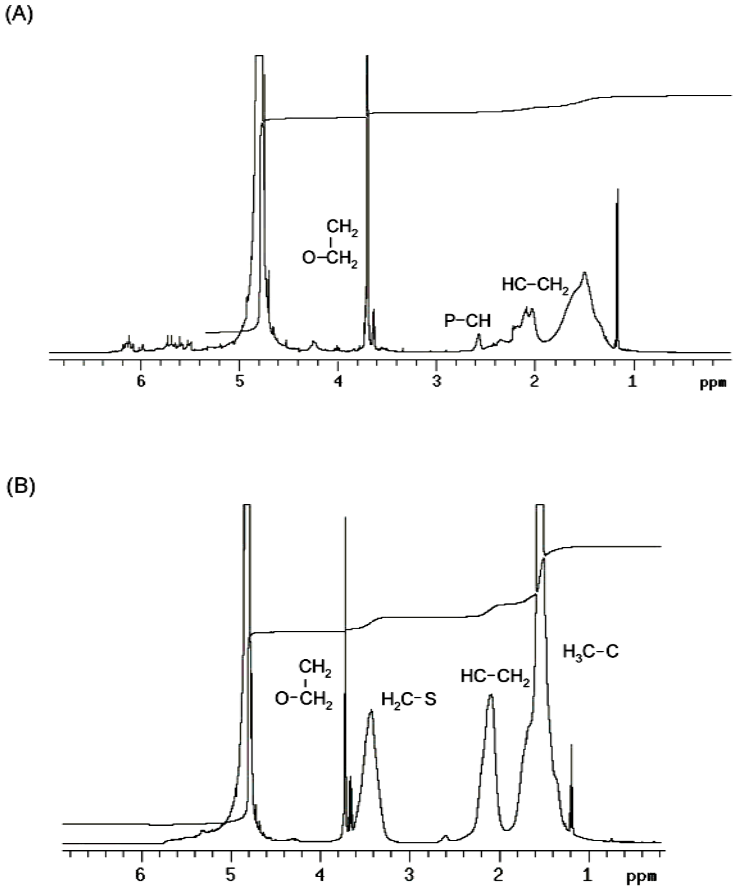

Figure S1. Representative H1-NMR of PCEs: (A) VPA20 and (B) AMPS30.

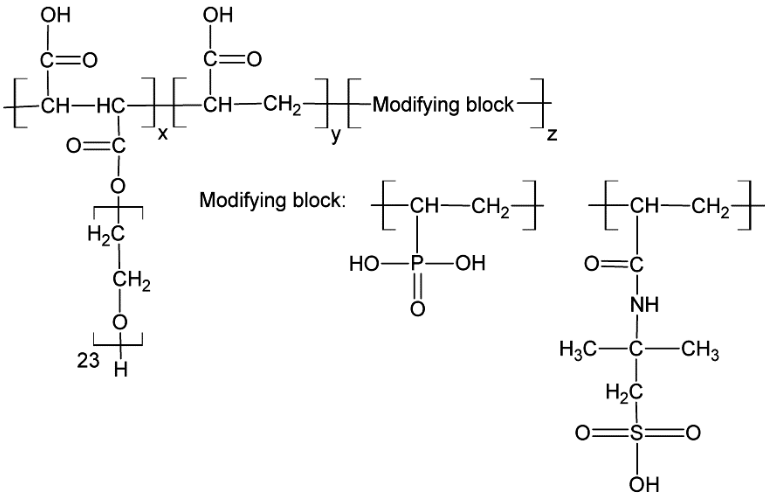

Figure S2. Chemical structure of PCEs.

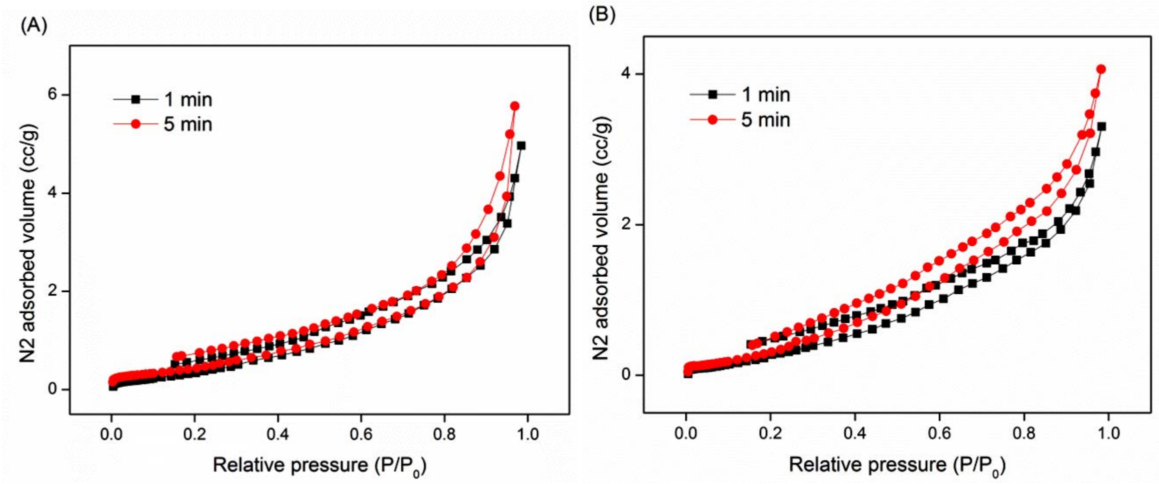

Figure S3. N_2 adsorption-desorption isotherm of (A) OPC and (B) CAC with hydration time.

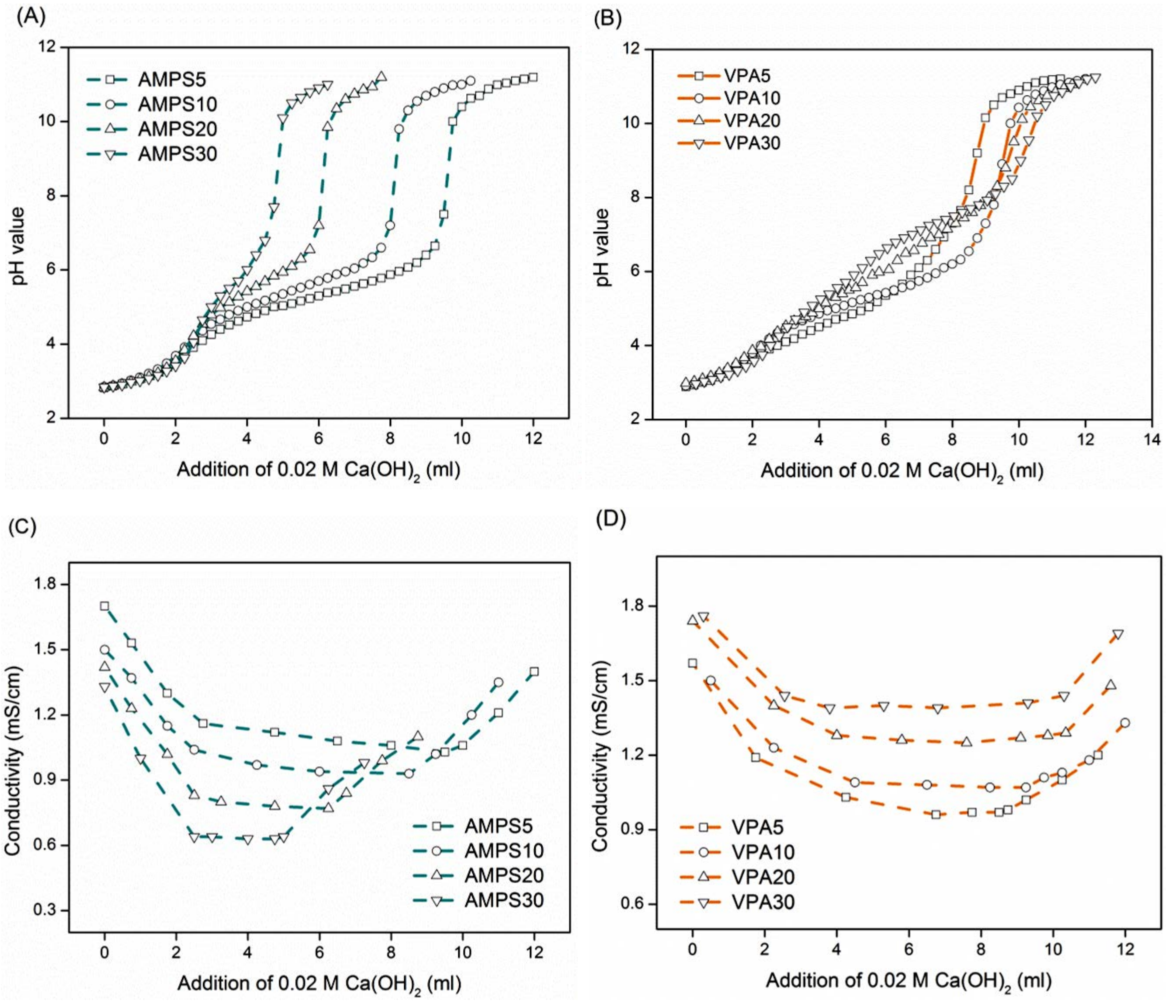

Figure S4. Ca-titration (A and B) and conductivity measurements (C and D) of PCEs.

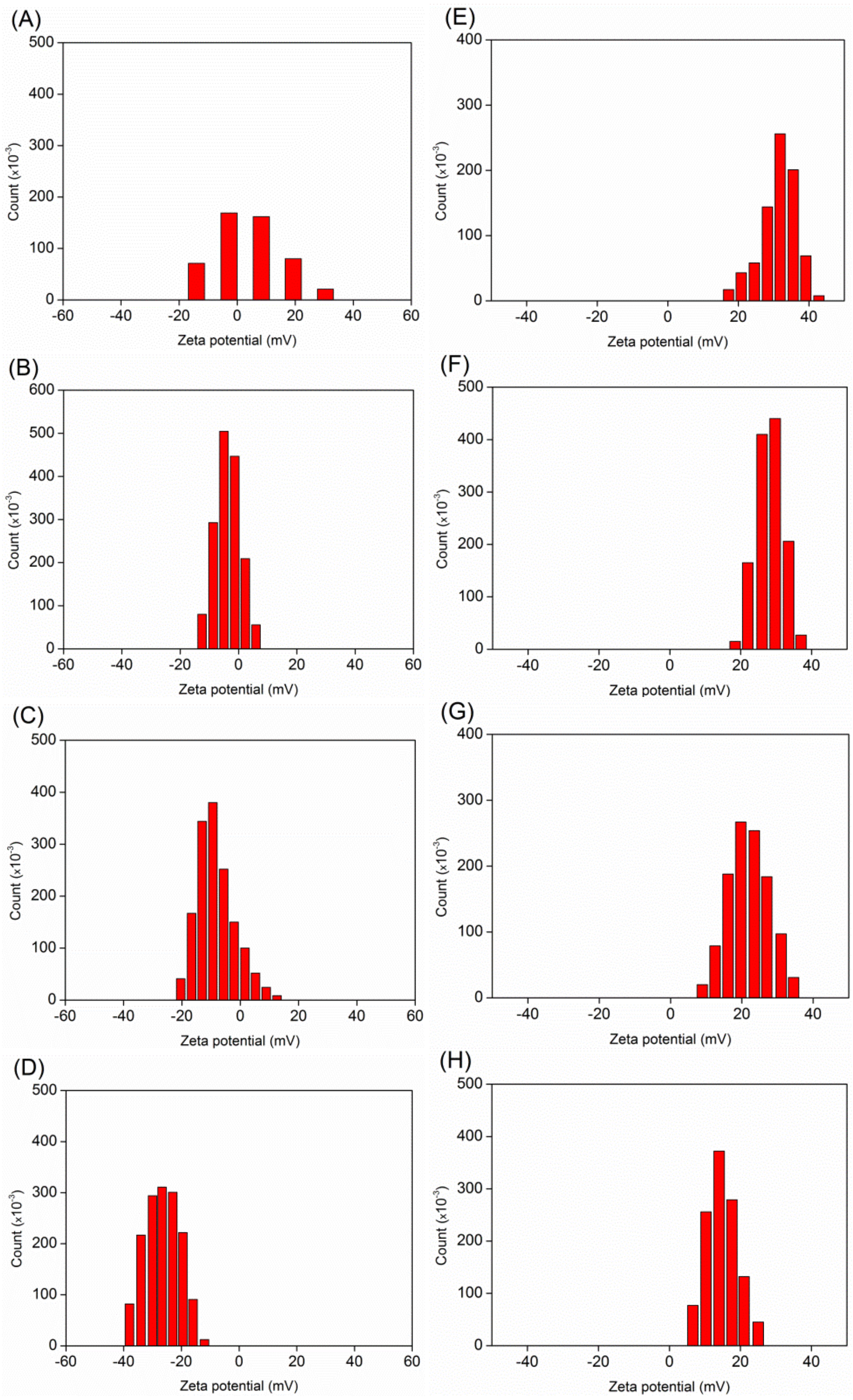

Figure S5. Zeta potential distribution of OPC-AMPS30 mixtures with PCEs content of (A) 0 wt%, (B) 0.05 wt%, (C) 0.1 wt%, (D) 0.2 wt%, and CAC-VPA30 mixtures with PCEs content of (E) 0 wt%, (F) 0.05 wt%, (G) 0.2 wt%, and (H) 0.4 wt%.

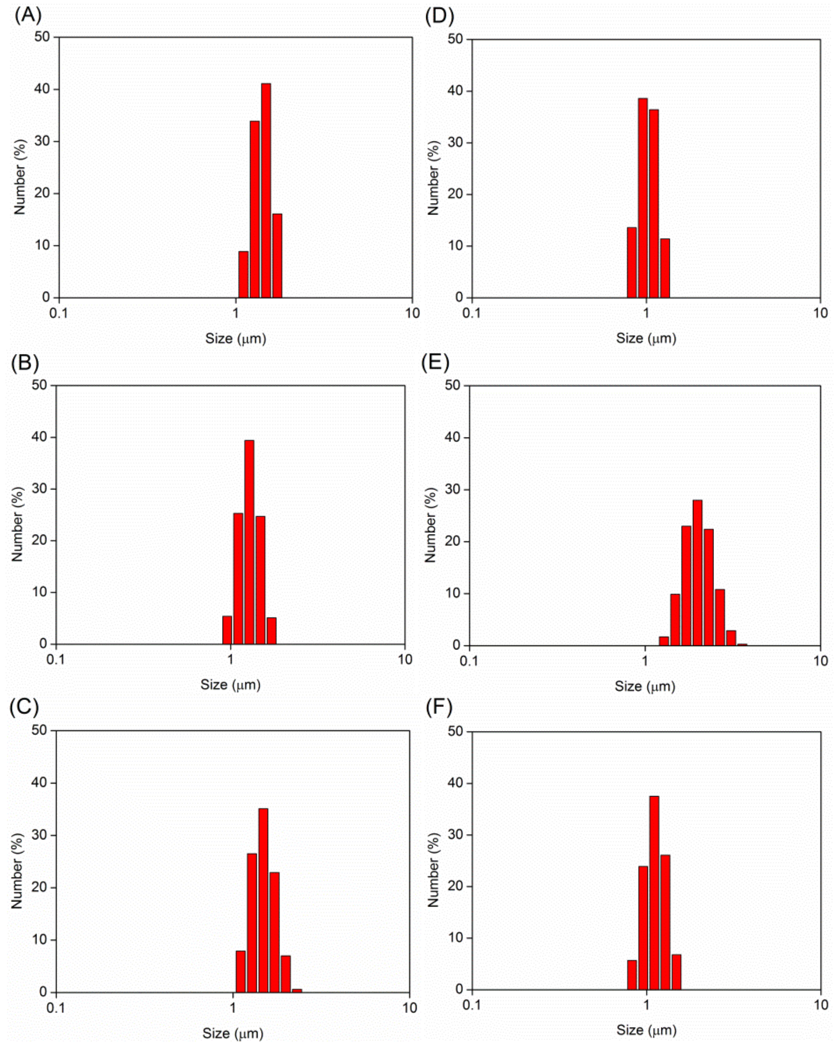

Figure S6. Size distribution of cement particles with and without 0.5 % bwoc PCEs: (A) neat OPC, (B) OPC-AMPS30, (C) OPC-VPA30, (D) neat CAC, (E) CAC-AMPS30, and (F) CAC-VPA30.

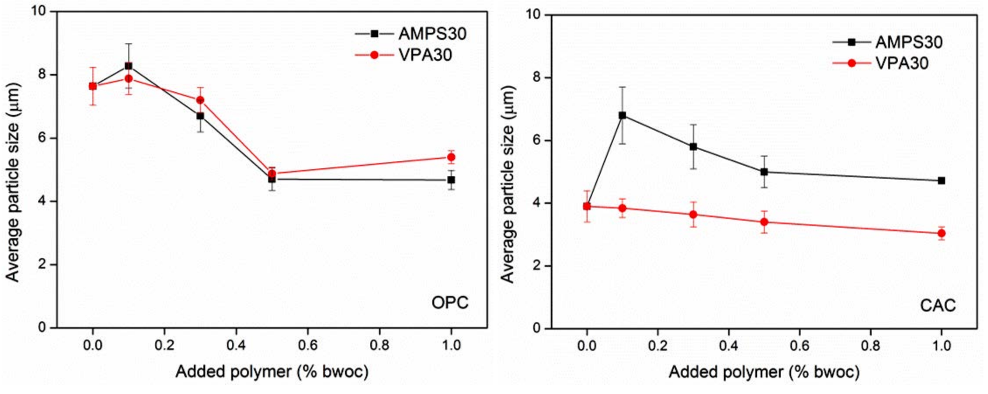

Figure S7. Effects of PCEs on the average particle size of OPC and CAC suspension.

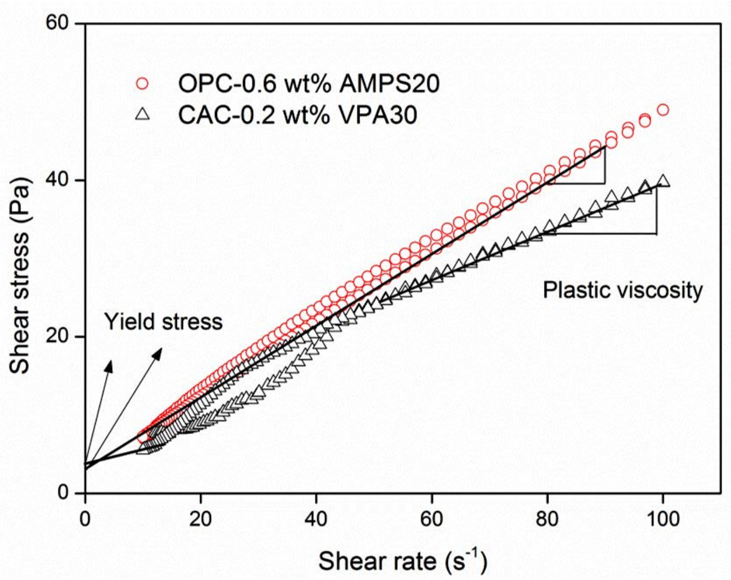

Figure S8. Hysteresis cycles with illustration of yield stress and plastic viscosity for cement pastes with admixtures.

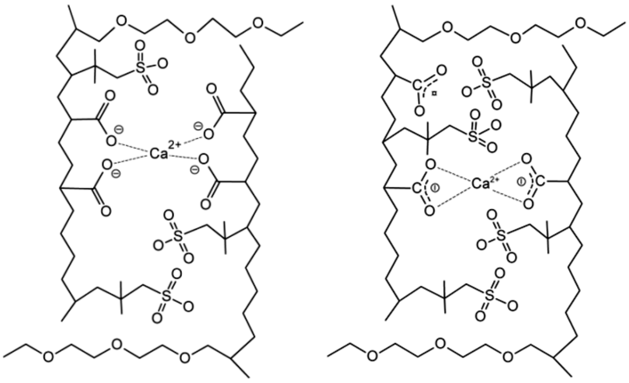

Figure S9. Schematic illustration of monodentate complexation of Ca^{2+} in PCEs with low AMPS/AA (left) and bidentate complexation of Ca^{2+} in PCEs with high AMPS/AA (right).

Table S1. Molar composition and characteristic properties of PCEs

	Molar feed ratio	Sample acronym	VPA/AA(1)	PEG/AA+VPA(1)	mmol anionic sites(2)/mg solid	mmol bound Ca(3)/mg solid
VPA/AA/PEG	5/45/1	VPA 5	0.8/10	1.1/100	4.1×10^{-3}	3.7×10^{-3}
	10/40/1	VPA 10	1.6/10	0.9/100	4.2×10^{-3}	3.9×10^{-3}
	20/30/1	VPA 20	3.4/10	1/100	4.4×10^{-3}	4.0×10^{-3}
	30/20/1	VPA 30	5.6/10	0.7/100	5.2×10^{-3}	4.2×10^{-3}
	Molar feed ratio	Sample acronym	AMPS/AA*	PEG/AA+AMPS*		
AMPS/AA/PEG	5/45/1	AMPS 5	3.1/10	0.6/100	4.6×10^{-3}	3.6×10^{-3}
	10/40/1	AMPS 10	4.2/10	0.5/100	5.6×10^{-3}	2.9×10^{-3}
	20/30/1	AMPS 20	7.5/10	0.5/100	5.8×10^{-3}	2.1×10^{-3}
	30/20/1	AMPS 30	17/10	0.7/100	6.2×10^{-3}	1.4×10^{-3}

(1) Determined by H^1-NMR
(2) Calculated from direct addition of NaOH to polymer solution by titration
(3) Calculated from direct addition of $Ca(OH)_2$ to polymer solution by titration

Table S2. BET surface area of hydrating cement

	BET surface area (m^2/g)		
	1 min hydration	3 min hydration	5 min hydration
Neat OPC	1.5	1.8	2.1
Neat CAC	1.8	2.1	2.3

Table S3. Polydispersity index of suspensions with and without 0.5 % bwoc PCEs

	OPC system	CAC system
Neat cement	0.80±0.1	0.53±0.02
AMPS-30	0.33±0.05	0.78±0.1
VPA-30	0.45±0.1	0.58±0.05

Table S4. Yield stress and plastic viscosity for OPC pastes and admixture dosage (n.d.: not determined due to severe flocculation)

	Dosage of PCEs (% bwoc)	Yield stress (Pa)	Plastic viscosity (Pa.s)
Pure OPC		14.4 ±1	0.5
AMPS10	0.2	10.1±0.5	0.58
	0.4	n.d.	n.d.
	0.6	n.d.	n.d.
AMPS20	0.2	7.7±0.4	0.90
	0.4	5.9±0.4	0.80
	0.6	5±0.2	0.67
AMPS30	0.2	14.2±1	0.49
	0.4	2.5±0.6	0.30
	0.6	2.1±0.5	0.35
VPA10	0.1	13±0.5	0.47
	0.2	14±0.7	0.58
	0.4	n.d.	n.d.
VPA20	0.1	13±0.3	0.41
	0.2	12±0.2	0.40
	0.4	11±0.2	0.39
VPA30	0.1	13.5±0.3	0.41
	0.2	12±0.3	0.41
	0.4	10.4±0.2	0.39

Table S5. Yield stress and plastic viscosity for CAC pastes and admixture dosage (n.d.: not determined)

	Dosage of PCEs (% bwoc)	Yield stress (Pa)	Plastic viscosity (Pa.s)
Pure CAC		7.8±0.3	0.30
VPA10	0.1	9.5±0.4	0.45
	0.2	11.1±0.5	0.41
	0.4	n.d.	n.d.
VPA20	0.1	7.5±0.3	0.36
	0.2	8.9±0.4	0.38
	0.4	14±0.5	0.40
VPA30	0.1	6.5±0.3	0.44
	0.2	5±0.2	0.46
	0.4	4.6±0.2	0.45

Table S6. Chemical analysis and physical properties of cements

Cement Type		OPC	CAC
Chemical composition (%)	SiO_2	13.37	3.60
	Al_2O_3	4.68	39.80
	Fe_2O_3	3.35	17.05
	CaO	63.08	36.20
	MgO	1.42	0.65
	SO_3	2.71	0.04
	Loss on ignition	3.30	0.30
Physical properties	Density (g/cm^3)	3.15	3.25
	Fineness (Blaine) (cm^2/g)	3275	3000
	BET surface area (m^2/g)	1.65	2.2
	Initial setting time	155	280
	Final setting time	250	295
	Residue in 45 µm sieve (%)	0	23
	Residue in 90 µm sieve (%)	0.3	6.5
Mechanical properties		2-day: 24.0 (MPa)<br>28-day: 52.6 (MPa)	6 Hours: 47 (MPa)<br>24 Hours: 70 (MPa)

Materials proportioning:

Different amounts of PCEs (0.5–10 mg/g cement) were first dissolved in water and then 100 g of cement grains were charged into these polymer solutions to make mixtures with water to cement ratio (w/c) of 0.3 in CAC and 0.4 in OPC system. All mixtures were prepared using high-shear mixer (Heidolph RZR 2102 equipped with a 4-blade stainless steel propeller) rotating at 1000 rpm for 5 min. Such a high-shear mixing regime was used to ensure good dispersion of cement grains and homogeneous suspensions. To perform the slump retention test, four different batches were prepared and then sealed to prevent the evaporation of water. In the intervals of 15, 30, 45, and 60 min after the first touch of water, labeled mixture was stirred at 300 rpm for 30 sec and slump test was conducted after 15 sec resting time of the mixture in the slump cone.

NaOH and $Ca(OH)_2$ titration:

Titration was conducted with a HI-2211 bench top pH meter on 50 ml solution of 1 mg/ml PCEs/water at 22 $\pm$ 2 °C. PCEs solutions were titrated with a 0.1 M NaOH and 0.02 M $Ca(OH)_2$.

Conductivity measurment:

Conductivity measurements were carried out with Zetasizer nanoseries (Malvern Instruments). PCEs solutions (1 mg/ml PCEs/water) were prepared and titrated with a 0.02 M $Ca(OH)_2$.

Determination of specific surface:

The Brunauer-Emmett-Teller (BET) specific surface area of samples was determined by analyzing the standard nitrogen adsorption isotherms at 77 K using nova 2200e, Quantachrome instruments. Suspensions with w/c of 0.4 (OPC) and 0.3 (CAC) were mixed for 1–5 min followed by centrifugation at 5000 rpm for 5 min. OPC and CAC particles were then redispersed in 2-propanol and acetone, respectively and centrifuged at 5000 rpm for 15 min. This process was repeated for 3 times following by vacuum drying at 40 °C for 3 days.

Characterization of dispersing state of cement suspensions:

Dispersing state of cement particles in aqueous medium was determined by dynamic light scattering (DLS) and measuring the Z-average (average particle size) of cement suspensions with and without polymers (Zetasizer nanoseries, Malvern Instruments, Ltd.). The suspensions with w/c of 40 and PCEs/cement of 0–10 mg/g were mixed by a magnetic stirrer for 10 min and then diluted by distilled water with a ratio of 1 to 20 (v/v). Each result represents the average value of 6 measurements, with 15 to 20 runs for each measurement.

Rheological measurements:

Rheological behavior of cement pastes was characterized by determining the plastic viscosity and yield stress using Anton-Paar MCR 302 rheometer equipped with cone-plate geometry of 50 mm/2° and a gap size of 0.208 mm at 25 ± 0.1 °C. All cement pastes were loaded immediately after cessation of the mixing process. After loading each sample, a thin layer of low viscosity paraffin oil (~150 mPa.s at 20 °C) was employed around the outer edge of the plates to protect the sample from evaporation. Rheological measurements were carried out by the measurement sequence as following; cement pastes were first kept under constant shear rate of 100 s^{-1} for 60 sec to ensure the structural breakdown. An increasing shear rate ramp from 10 to 100 s^{-1} in 150 sec was then applied followed by a decreasing ramp rate from 100 s^{-1} to 10 s^{-1} in 150 sec. The flow curve of descending shear rates was modeled using Bingham equation ($\tau = \tau_0 + \eta\gamma$) where τ is the shear stress (Pa), τ_0 is the yield stress (Y-intercept, Pa), and η is plastic viscosity (Pa.s). When pastes are totally deflocculated, demonstrated by no hysteresis cycle, they behaved like Bingham fluids and the shear rate descent curves could be fitted to the Bingham equation (OPC-0.6 wt% AMPS20 in Figure S8). In some cases, the relationship between shear rate and shear stress was not linear. Therefore, plastic viscosity and shear yield stress were assessed from the relationship between shear stress and shear rate as shown by 0.2 wt%VPA30 in Figure S8. This kind of non-linear behavior was also reported by Yamada et al. [2] Here, shear yield stress and plastic viscosity were defined as the intercept

point of the linear part nearest the Y-axis and the slope of the linear part of the relationship around 50 to 100 s^{-1}, respectively.

Characterization of zeta potential of suspensions in plateau region:

Apart from the nature of bonding of co-monomers and cement particles, electro-kinetic behaviors of PCEs in cement types depends on steric accessibility of COO^{-} groups of the main monomer (AA) in the backbone. The carboxylate groups in PCEs provide two types of Ca^{2+} mediated adsorption: i) monodentate ligand (Figure S9A) and ii) bidentate ligand (Figure S9B), which shows no anionic charge in pore solution. When carboxylate groups are shielded by other functionalities (e.g., VPA and bulky AMPS co-monomer), they adopt bidentate conformation and hence, anionicity of the PCEs declines.[1] This shielding effect supports the lower zeta potential of OPC suspensions when PCEs with highest content of co-monomers (Table S2) are added to the system (AMPS30 and VPA30 compared to AMPS20 and VPA20, respectively in Figure 2C and 2D).

[1] J. Plank, B. Sachsenhauser, Cement and Concrete Research 2009, 31, 1.
[2] K. Yamada, T. Takahashi, S. Hanehara, M. Matsuhisa, Cement and Concrete Research 2000, 30, 197.
